# Spontaneous cortical activity is altered in persons with HIV and related to domain-specific cognitive function

**DOI:** 10.1093/braincomms/fcae228

**Published:** 2024-07-13

**Authors:** Nathan M Petro, Maggie P Rempe, Mikki Schantell, Vivian Ku, Advika N Srinivas, Jennifer O’Neill, Maureen E Kubat, Sara H Bares, Pamela E May-Weeks, Tony W Wilson

**Affiliations:** Institute for Human Neuroscience, Boys Town National Research Hospital, Boys Town, NE 68010, USA; Institute for Human Neuroscience, Boys Town National Research Hospital, Boys Town, NE 68010, USA; College of Medicine, University of Nebraska Medical Center (UNMC), Omaha, NE 68198, USA; Institute for Human Neuroscience, Boys Town National Research Hospital, Boys Town, NE 68010, USA; College of Medicine, University of Nebraska Medical Center (UNMC), Omaha, NE 68198, USA; Institute for Human Neuroscience, Boys Town National Research Hospital, Boys Town, NE 68010, USA; Institute for Human Neuroscience, Boys Town National Research Hospital, Boys Town, NE 68010, USA; Department of Internal Medicine, Division of Infectious Diseases, UNMC, Omaha, NE 68198, USA; Department of Internal Medicine, Division of Infectious Diseases, UNMC, Omaha, NE 68198, USA; Department of Internal Medicine, Division of Infectious Diseases, UNMC, Omaha, NE 68198, USA; Department of Neurological Sciences, UNMC, Omaha, NE 68198, USA; Institute for Human Neuroscience, Boys Town National Research Hospital, Boys Town, NE 68010, USA; Department of Pharmacology and Neuroscience, Creighton University, Omaha, NE 68178, USA

**Keywords:** neuroHIV, cognitive domains, magnetoencephalography, MEG, resting state

## Abstract

Whilst the average lifespan of persons with HIV now approximates that of the general population, these individuals are at a much higher risk of developing cognitive impairment with ∼35–70% experiencing at least subtle cognitive deficits. Previous works suggest that HIV impacts both low-level primary sensory regions and higher-level association cortices. Notably, multiple neuroHIV studies have reported elevated levels of spontaneous cortical activity during the pre-stimulus baseline period of task-based experiments, but only a few have examined such activity during resting-state conditions. In the current study, we examined such spontaneous cortical activity using magnetoencephalography in 79 persons with HIV and 83 demographically matched seronegative controls and related this neural activity to performance on neuropsychological assessments of cognitive function. Consistent with previous works, persons with HIV exhibited stronger spontaneous gamma activity, particularly in inferior parietal, prefrontal and superior temporal cortices. In addition, serostatus moderated the relationship between spontaneous beta activity and attention, motor and processing speed scores, with controls but not persons with HIV showing stronger beta activity with better performance. The current results suggest that HIV predominantly impacts spontaneous activity in association cortices, consistent with alterations in higher-order brain function, and may be attributable to deficient GABAergic signalling, given its known role in the generation of gamma and beta oscillations. Overall, these effects align with previous studies showing aberrant spontaneous activity in persons with HIV and provide a critical new linkage to domain-specific cognitive dysfunction.

## Teaser

Petro et *al*. report that people with HIV compared with controls exhibit stronger spontaneous gamma activity in frontotemporal cortices. In controls only, stronger beta activity scaled with better cognitive performance in the motor, attention and processing speed domains. Thus, spontaneous cortical activity was aberrant in people with HIV.

## Graphical Abstract

**Graphical Abstract:**
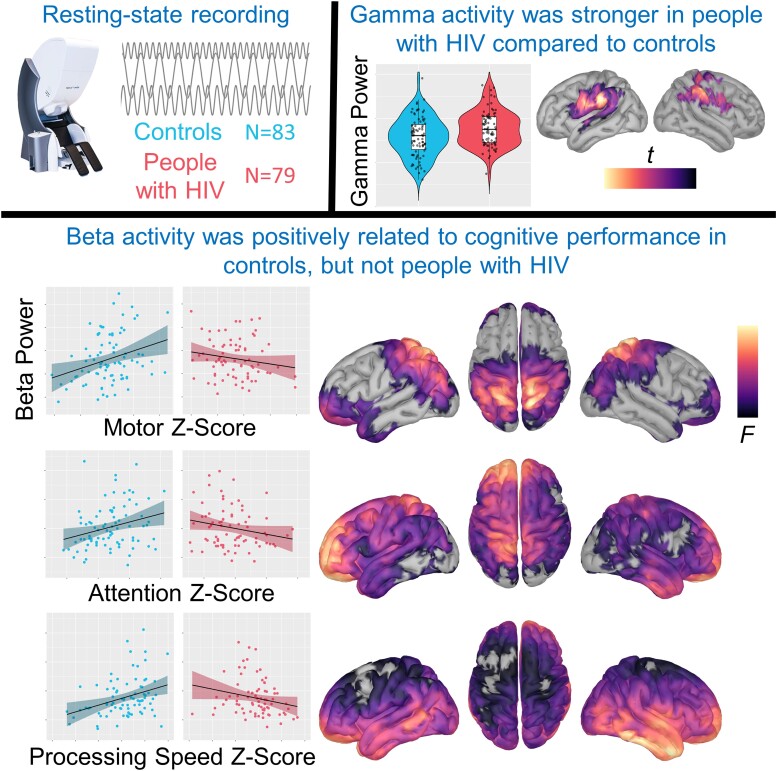

## Introduction

Combination antiretroviral therapy (ART) has dramatically increased the life expectancy of persons with HIV (PWH), which now approximates that of healthy controls.^1,2^ Whilst the effects of ART have also decreased the incidence of severe cognitive decline,^3^ ∼35–70% of PWH still exhibit at least subtle impairments on neuropsychological assessments, which has been termed HIV-associated neurocognitive disorder (HAND).^4-8^ Generally, three different levels of HAND are recognized, with asymptomatic neurocognitive impairment being the mildest, followed by mild neurocognitive disorder and HIV-associated dementia being the most severe.^4,9,10^ Across all levels of severity, those with HAND are more likely to be unemployed or underemployed, have greater problems with medication adherence and have lower quality of life.^11-14^ Thus, developing neurotherapeutics remains an important goal, and identifying the underlying neural mechanisms is critical to these efforts.

Both low-level sensory processes and higher-order functioning are impacted by HIV, which is able to cross the blood–brain barrier and is thought to instigate widespread neuroinflammation.^15,16^ Previous studies have shown reduced cortical thickness in somatosensory and visual cortices in PWH^17^ and aberrant activation in these regions during somatosensation^18-20^ and visual attention tasks,^21-23^ respectively. In addition, other works have shown that PWH exhibit disrupted activation in the frontoparietal attention network during visual attention tasks,^24-31^ suggesting that deficits may also arise within higher level brain systems. Along these lines, studies have also shown widespread alterations in grey matter thickness and volume,^32-34^ as well as white matter integrity.^35,36^ Thus, taken together, the current findings indicate that HIV affects multiple brain areas, including low-level sensory cortices and higher-order regions and networks.

Whilst considerable progress has been made in identifying brain aberrations in PWH using structural imaging and task-based and resting-state functional MR, very few studies have investigated spontaneous cortical activity. Briefly, neurons in the human cerebral cortex are known to exhibit spontaneous discharges and fluctuations in dendritic currents, as well as other electrical field activities even in the absence of endogenous and exogenous inputs.^37^ These neural phenomena locally summate and give rise to population-level rhythms that are generally known as ‘spontaneous activity’. Such spontaneous rhythms are ubiquitous in the human brain but vary in amplitude and spectral content depending on the behavioural state of the person, the activation state of the ensemble, the specific brain area and other parameters such as age. Measuring spontaneous activity requires tools with high temporal resolution (i.e. millisecond level) and can be considerably challenging. Amongst available modalities, magnetoencephalography (MEG) is particularly well suited, as the method provides both high temporal resolution and good spatial precision, thus enabling functional maps of spontaneous cortical activity to be computed and examined for HIV-related effects.

Early work showed that elevated spontaneous activity was associated with mental fatigue in young adults^38^ and that spontaneous activity increased in healthy ageing.^39-41^ However, the most recent data suggest that there is some spectral specificity with lower frequency spontaneous activity (e.g. delta and theta) decreasing with age and higher frequency (e.g. alpha, beta and gamma) increasing with age.^42^ In addition, multiple studies have shown that spontaneous alpha and gamma activity is elevated in PWH compared with controls, which, given the findings noted above, may further support the notion of accelerated ageing in PWH.^30,33,43,44^ Such elevated spontaneous activity has been shown across multiple brain regions, including those serving visual attention,^22,45^ somatosensation^18-20^ and working memory.^46^ Furthermore, some work has shown that the strength of spontaneous cortical activity can also distinguish cognitively impaired PWH from those who are unimpaired.^22,28^ Whilst these results present a promising biomarker for cognitive decline in PWH, such measures of spontaneous activity in PWH have been limited to the baseline period (i.e. pre-stimulus intervals) of task-based experiments. The two studies that have measured resting-state spontaneous activity in PWH^47,48^ did not report elevated activity, but the findings across the two studies were inconsistent, potentially due to the sample sizes that were used. Thus, additional studies in this area are clearly warranted.

Herein, we used an advanced multispectral MEG imaging approach to measure differences in spontaneous cortical activity during rest in PWH compared with controls. First, we predicted that PWH would show elevated spontaneous cortical activity, particularly in the gamma band, consistent with several previous task-based studies that focussed on the baseline period. In addition, we examined whether the neuropsychological assessments used to identify cognitive decline in PWH were related to particular patterns of spontaneous cortical activity. Specifically, we tested whether individual domains of neuropsychological function were indicative of aberrant spontaneous activity in a specific brain region and whether this relationship differed between PWH and controls. Lastly, we probed the effect of HAND status on these relationships by testing if those with HAND were the drivers of any observed effects.

## Materials and methods

### Participants

**Figure 1:**
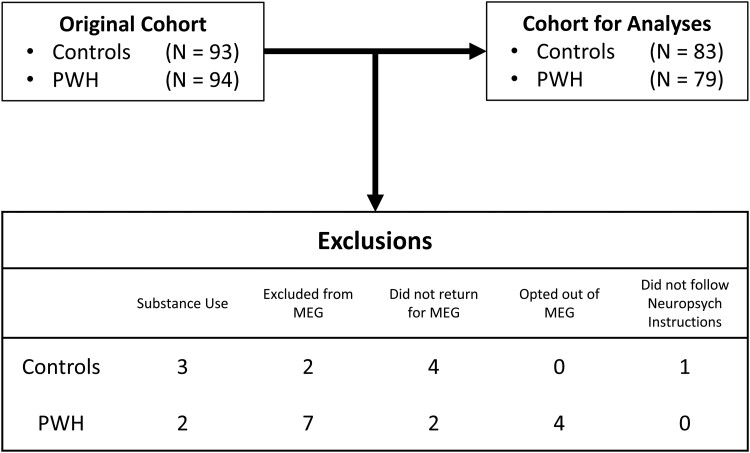
**CONSORT diagram.** Of the 187 participants enrolled in the study, 162 remained following exclusion for current substance use, MEG incompatibility, failure to return for the MEG session, opting out of the MEG session and failure to follow instructions during neuropsychological assessment.

A total of 187 participants (mean: 46.21 years; SD: 12.94; minimum: 20 years; maximum: 75 years) were enrolled in this study. Of these, 94 were virally suppressed seropositive participants (mean: 47.08 years; SD: 12.87 years) and 93 were seronegative controls (mean: 45.35; SD: 13.03). Of these, six (two PWH) did not return for their MEG recording, five (two PWH) were excluded for substance use, four PWH opted out of the study after enrolment, nine (seven PWH) were excluded from MEG recording based on standard exclusion criteria, and one control was excluded for failure to follow the instructions of the neuropsychological assessments (Fig. 1). Thus, 162 participants (mean: 46.43 years; SD: 13.31 years; minimum: 20 years; maximum: 75 years) completed this study. This sample size is consistent with recent studies showing medium to large effect sizes when testing individual differences in MEG activity of controls and PWH.^21,29^ Of these, 79 were virally suppressed seropositive participants (mean: 47.32 years; SD: 13.01 years), and 83 were seronegative (mean: 45.59 years; SD: 13.62 years). PWH were eligible if on ART with an HIV-1 RNA viral load of <50 copies/ml within 3 months of participation in the study. All controls were confirmed to be seronegative for HIV using the OraQuick *ADVANCE*® Rapid HIV-1/2 Antibody Test^49^ at the time of neuropsychological testing. The two groups were age and sex matched to prevent any confounding effects (see Table 1). Exclusionary criteria included any medical illness affecting the CNS, any neurological or psychiatric disorder, history of head trauma, current substance use disorder and standard MEG exclusion criteria (e.g. ferromagnetic implants). The local Institutional Review Board approved the study protocol. Written informed consent was obtained from each participant after a full description of the study. All study procedures took place between 2019 and 2020.

**Table 1:** Participant demographics and neuropsychological profiles

		HIV (*n* = 79)	Controls (*n* = 83)	*P*-value
**Demographics**	**Age (years)**	47.315 (13.006)	45.588 (13.621)	0.411
	**Sex assigned at birth (*n*)**	61 male/18 female	66 male/17 female	0.72
	**Race (*n*)**	61 White/9 Black/3 Asian/1 Native American/5 Multiracial	66 White/8 Black/6 Asian/0 Native American/3 Multiracial	0.62
	**Ethnicity (*n*)**	68 not Hispanic/9 Hispanic/2 NA	77 not Hispanic/6 Hispanic	0.22
	**Education (years)**	13.872 (2.467)	16.313 (2.06)	<0.001
**HIV clinical metrics**	**CD4 nadir (cells/µl)**	243 (215.772)		
	**Current CD4 count (cells/µl)**	782.029 (374.484)		
	**current viral load (copies/ml)**	<50^a^		
	**Years since HIV diagnosis**	14.215 (8.886)		
	**Years on ART**	12.329 (8.059)		
**Neuropsychological performance**	**Learning *Z*-score**	0.307 (0.969)	0.723 (0.761)	0.005
	**Memory *Z*-score**	0.312 (0.894)	0.726 (0.739)	0.005
	**Processing speed *Z*-score**	−0.119 (0.829)	0.122 (0.958)	0.11
	**Attention *Z*-score**	−0.108 (0.718)	0.383 (0.721)	<0.001
	**Executive function *Z*-score**	0.101 (0.914)	0.403 (0.994)	0.069
	**Language *Z*-score**	−0.027 (0.921)	0.023 (0.872)	0.72
	**Motor *Z*-score**	−0.333 (0.845)	0.071 (0.85)	0.005

Means and standard deviations are displayed for each continuous variable. Differences in mean age between males and females were assessed using an independent samples *t*-test; differences in race, ethnicity and handedness were assessed using *χ*^2^ tests. *P*-values for neuropsychological performance variables are FDR corrected.

ART, antiretroviral therapy; NA, not answered.

^a^Participants with a viral load of >50 copies/ml were ineligible to participate in this study.

### Neuropsychological testing

All participants performed a neuropsychological assessment battery that aligned with the Frascati consensus.^4^ This test battery assessed seven cognitive domains, consisting of ‘motor dexterity’ (Grooved Pegboard, dominant and non-dominant HANDs^50,51^), ‘language’ (phonemic verbal fluency and semantic verbal fluency^50^), ‘learning’ (Wechsler Memory Scale Logical Memory,^52^ California Verbal Learning Test II Learning Trials 1–5^53^), ‘processing speed’ (Comalli Stroop Test Color and Word Trials,^54^ Wechsler Adult Intelligence Scale III Digit Symbol Coding,^55^ Trail Making Part A^50^), ‘attention’ (Wechsler Adult Intelligence Scale III Letter Number Sequencing,^55^ Wechsler Adult Intelligence Scale III Digit Span Forward and Backward Trials,^55^ California Verbal Learning Test II Trial 1^53^), ‘memory’ (California Verbal Learning Test II Delayed Recall and Recognition Discriminability Index, Wechsler Memory Scale III Logical Memory II Delayed Recall^52^) and *‘*executive functioning’ (Comalli Stroop Test Interference Trial,^54^ Trail Making Test Part B^50^). Each assessment score was demographically corrected (including age) using published normative data^50-55^ and transformed into to *Z*-scores. Composite scores for each of these cognitive domains were computed by averaging the resulting *Z*-scores from the assessment sets comprising each domain. The HAND status was assigned per a modified approach to the Frascati criteria^4^ by a clinical neuropsychologist and research assistant. To determine if PWH differed from controls in any of the neuropsychological assessment domains, each composite score was submitted to an independent samples *t*-test. The executive functioning and processing speed scores were discarded for four participants due to the inability to complete the Stroop task. The motor dexterity score was discarded for two participants due to the inability to complete the Grooved Pegboard task. These participants were removed from the respective analyses involving executive function, processing speed and motor dexterity scores.

### MEG data acquisition

MEG recordings were conducted in a one-layer magnetically shielded room with active shielding engaged to compensate for environmental disturbances. Neuromagnetic responses were recorded continuously at a 1-kHz sampling rate using an acquisition bandwidth of 0.1–330 Hz and a MEGIN MEG system equipped with 204 planar gradiometers and 102 magnetometers. During the 6-min recording, participants were seated with their eyes closed and were monitored by a real-time audio-visual system to ensure compliance.

### Structural MRI acquisition, processing and MEG-MRI co-registration

Individual structural MRI data for each participant were acquired using a Siemens Prisma 3T scanner (Siemens Medical Solutions) with a 64-channel head coil and an MP-RAGE sequence with the following parameters: Repitition time = 2300 ms; Echo time = 2.98 ms; flip angle = 9°; Field-of-view = 256 mm; slice thickness = 1.00 mm; and voxel size = 1 × 1 × 1 mm. All T_1_-weighted structural MRI data were segmented with the computational anatomy toolbox (CAT12 v12.6^56^) within SPM12. Here, segmented T_1_ images underwent noise reduction using a spatially adaptive non-local means denoising filter^57^ and a classical Markov random field approach.^58^ An affine registration and a local intensity transformation were then applied to the bias-corrected images. These pre-processed images were segmented based on an adaptive maximum *a posteriori* technique^59^ and a partial volume estimation with a simplified mixed model of a maximum of two tissue types. Lastly, the segmented images were normalized to Montreal Neurological Institute template space and imported into Brainstorm for co-registration.

Prior to MEG acquisition, four coils were attached to the participants’ heads and localized, together with the three fiducial points and scalp surface, using a 3D digitizer (Fastrak, Polhemus Navigator Sciences, Colchester, VT, USA). Once positioned in the MEG, the coils were driven with an electrical current at a unique frequency, and the magnetic fields associated with these currents were localized in reference to the MEG sensor array. Since coil locations were also known in head coordinates, all MEG measurements could be transformed into a common coordinate system. With this coordinate system, each participant's MEG data were co-registered with structural T_1_-weighted MRI data prior to source space analysis using Brainstorm (see the ‘MEG source imaging and frequency power maps’ section).

### MEG data preprocessing

Each set of MEG data was individually corrected for head motion and underwent noise reduction using the signal space separation method with a temporal extension (tSSS; MaxFilter v2.2; correlation limit: 0.950; correlation window duration: 6 s^60^). These noise-reduced MEG data then underwent standard data pre-processing using the Brainstorm software. This process included a high pass filter of 1 Hz, a low pass filter of 200 Hz and a notch filter of 60 Hz and its harmonics to eliminate line noise. Cardiac artefacts were identified and removed using signal-space projection, which was subsequently accounted for during source reconstruction.^61,62^ Data were split into 4-s epochs for the detection and rejection of bad segments of MEG data. Amplitude and gradient metrics for each 4-s epoch were computed using custom MATLAB code, and epochs that contained outliers were rejected using a standardized fixed threshold of three standard deviations. Following this artefact rejection step, the mean number of accepted epochs was 78.52 (SD = 11.74, min = 57, max = 162). The total number of accepted epochs did not statistically differ between controls and PWH.

### MEG source imaging and frequency power maps

The procedures used to source image MEG data followed the analysis pipeline outlined by Wiesman *et al*.^63^ Using an overlapping sphere model that is not constrained to the cortical surface,^64^ this analysis models a single sphere for each of the 204 gradiometers (minus any channels previously labelled as bad). A linearly constrained minimum variance beamformer was then used to spatially filter the data based on the data covariance, computed from the resting-state recordings, and the noise covariance, computed from empty room recordings. Note, except for the removal of cardiac artefacts, the empty room recordings underwent pre-processing steps identical to the recordings of spontaneous cortical activity.

The resulting data were used to estimate the power of cortical activity in each of the canonical frequency bands (delta: 2–4 Hz; theta: 5–7 Hz; alpha: 8–12 Hz; beta: 15–29 Hz; low gamma: 30–59 Hz; high gamma: 60–90 Hz). Using Welch's method,^65^ power spectrum densities were estimated for each 4-s epoch across each MEG recording, with a 1-s sliding Hamming window that overlapped at 50%. These raw power spectrum density maps were averaged across epochs to obtain one absolute power spectrum density map per participant, with units corresponding to the Pseudo Neural Activity Index, which is a modified version of Van Veen's Neural Activity Index.^66^ Lastly, the norm of the three unconstrained orientations per location was then projected onto a common Montreal Neurological Institute ICBM152 brain template^67^ surface, and a 3-mm full width at half maximum smoothing kernel was applied before further statistical analysis.

### Whole-brain statistical analysis of MEG data

Statistical analysis of MEG data was conducted in SPM12. Whole-brain differences in spontaneous oscillatory activity between PWH and controls were assessed using vertex-wise independent samples *t*-test. This yielded a statistical map of *t*-values per canonical band representing the group difference between PWH and controls. The relationship between spontaneous neural dynamics, HIV and cognitive assessment scores was assessed using vertex-wise multiple regression. To this end, the spontaneous power during rest, separately for each vertex, was submitted as the outcome in a multiple regression across subjects with the predictors of cognitive score, serostatus and their interaction; this process was repeated separately for each frequency band and cognitive domain. The coefficients from the interaction term were submitted to a contrast, producing an *F*-value representing the interaction effect.

The resulting statistical cortical maps from the *t*-test and regression were then subjected to threshold-free cluster enhancement (*E* = 1, *H* = 2; 5000 permutations), a non-parametric approach to determine cluster- and vertex-based thresholds.^68^ Following the permutation tests, these threshold-free cluster enhancement maps were assessed with a cluster-wise threshold of *P*_fwe_ < 0.05 and a cluster forming threshold of *k* > 100 vertices.

## Results

### Neuropsychological outcomes

**Figure 2:**
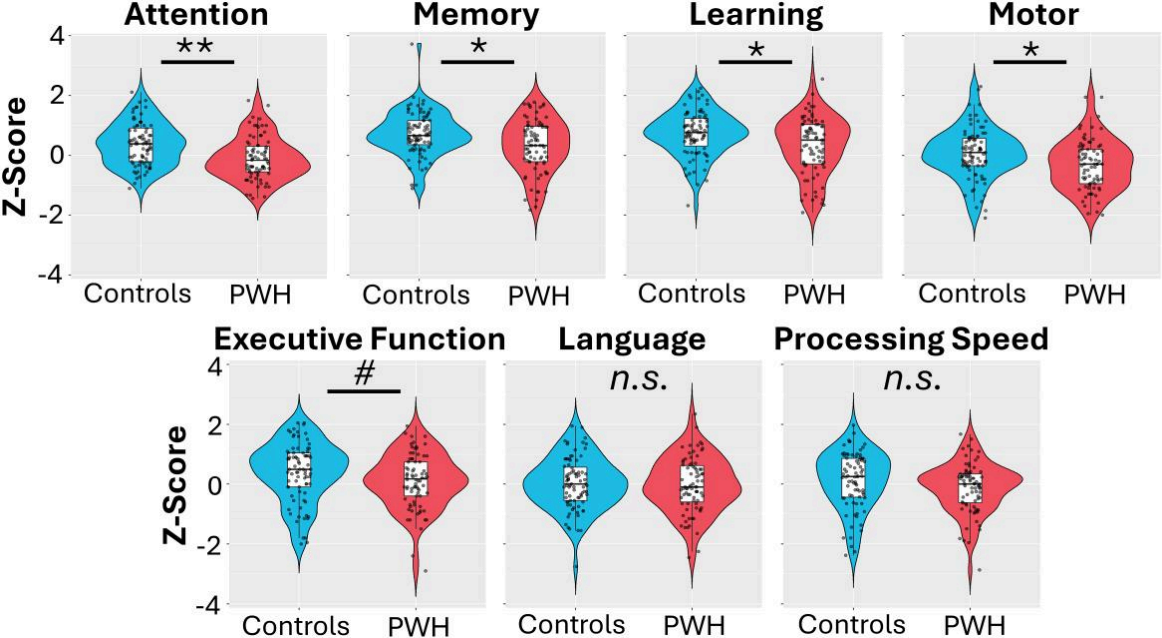
**Group differences in the composite scores for neuropsychological assessments.**  *Z*-scores for each domain of cognitive assessment for both controls and PWH. Dots represent the *Z*-score for each participant. The box plots illustrate the mean, first and third quartiles, and the whiskers indicate the minima and maxima. The violin plots illustrate the probability density. As revealed in independent samples *t*-tests, controls scored higher on the attention (*t*_160_ = 4.34, *P*_fdr_ < 0.001), memory (*t*_160_ = 3.22, *P*_fdr_ < 0.01), learning (*t*_160_ = 3.05, *P*_fdr_ < 0.01) and motor (*t*_158_ = 3.01, *P*_fdr_ < 0.01) domains relative to PWH (**P_fdr_ < 0.001, *P_fdr_ < 0.01, #*P* = 0.069). Controls and PWH did not differ in the executive function, language or processing speed domains.

We tested whether PWH differed from controls on any of the neuropsychological assessments using independent samples *t*-test. PWH performed worse than controls on the fine motor (*t*_158_ = 3.01, *P*_fdr_ < 0.01), attention (*t*_160_ = 4.34, *P*_fdr_ < 0.001), memory (*t*_160_ = 3.22, *P*_fdr_ < 0.01) and learning (*t*_160_ = 3.05, *P*_fdr_ < 0.01) scores (see Fig. 2). There were no differences between controls and PWH on the language, executive function or processing speed scores (all *P*s > 0.05).

### Effect of HIV serostatus on spontaneous cortical activity

**Figure 3:**
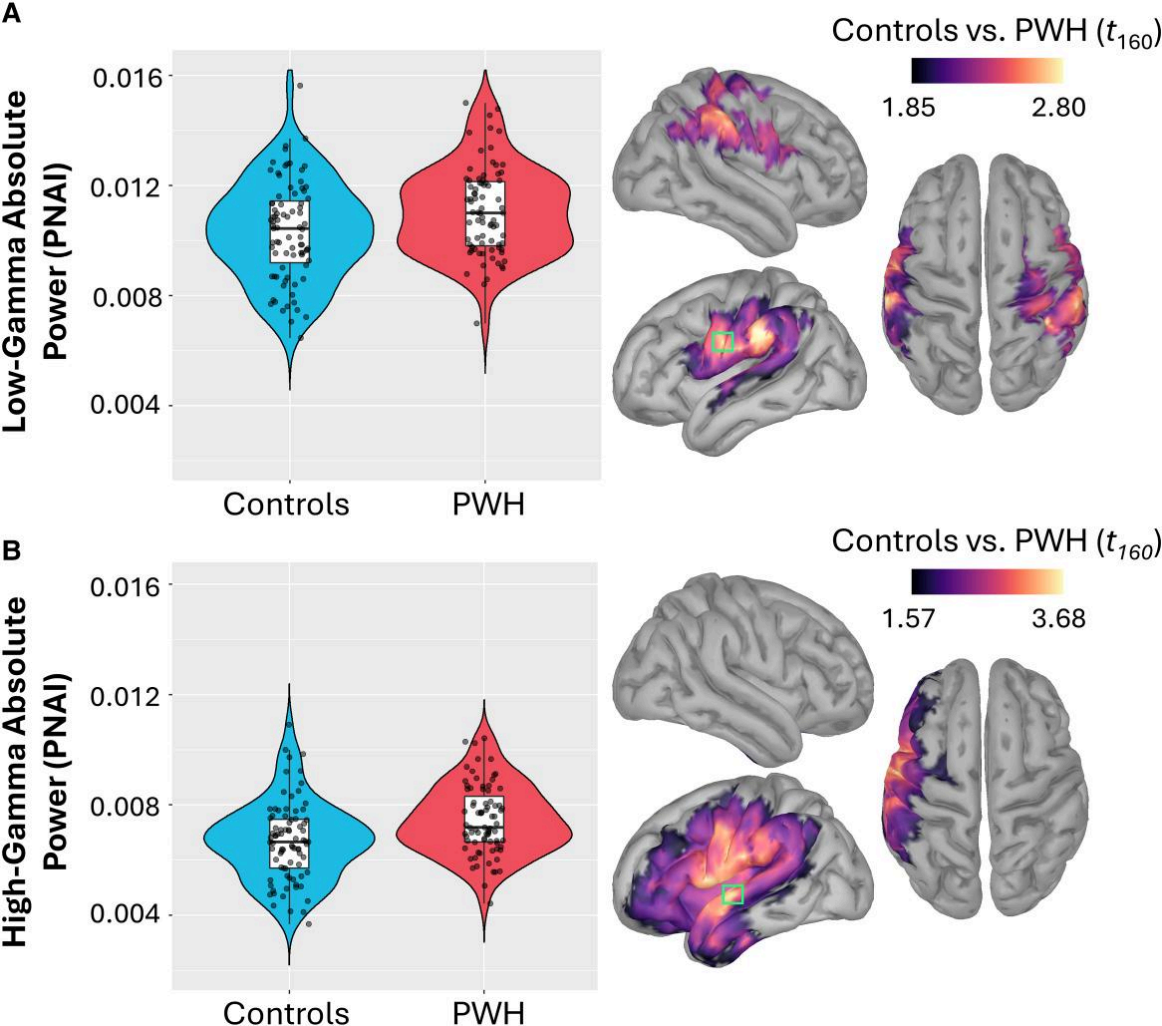
**Group differences in resting spontaneous gamma power.** Cortical surface maps (right) display the vertex-wise *t*-values from independent samples *t*-tests of the group comparison of spontaneous power for low-gamma (**A**) and high-gamma (**B**) power. The green box indicates the vertex containing the strongest difference. For illustrative purposes, each participant's absolute power at the peak is plotted (left) separately for controls and PWH. The box plots illustrate the mean, first and third quartiles, and the whiskers indicate the minima and maxima. The violin plots illustrate the probability density. PWH had stronger power for both low-gamma (*t*_160_ = 2.80, *P*_fwe_ < 0.05) and high-gamma (*t*_160_ = 3.68, *P*_fwe_ < 0.05) compared with controls, with differences being generally confined to inferior parietal, prefrontal and superior temporal cortices.

The difference in spontaneous cortical activity between PWH and controls was assessed using independent samples *t*-tests, conducted across each vertex. Group differences were observed in the low- and high-gamma bands, such that PWH exhibited stronger spontaneous activity in these frequency bands. In low-gamma range, this effect peaked in a lateral region of the left central sulcus (*t*_160_ = 2.80, *P*_fwe_ < 0.05; Fig. 3A) and included coverage of both the pre- and post-central gyri bilaterally, as well as the bilateral supramarginal gyri and the left superior temporal gyrus. In the high-gamma range, this effect involved similar anatomical regions with the addition of the prefrontal cortices but was strongly lateralized to the left hemisphere and peaked in the left superior temporal gyrus (*t*_160_ = 3.68, *P*_fwe_ < 0.05; Fig. 3B). No group differences in spontaneous activity were found in the delta, theta, alpha or beta spectral bands.

### Effect of HIV on the relationship between cognitive performance and spontaneous activity

We examined the moderating effect of HIV serostatus on the relationship between cognitive domain performance and spontaneous cortical activity using vertex-wise regression. This analysis revealed moderating effects on the relationship between spontaneous beta activity and the attention, motor and processing speed domains.

**Figure 4:**
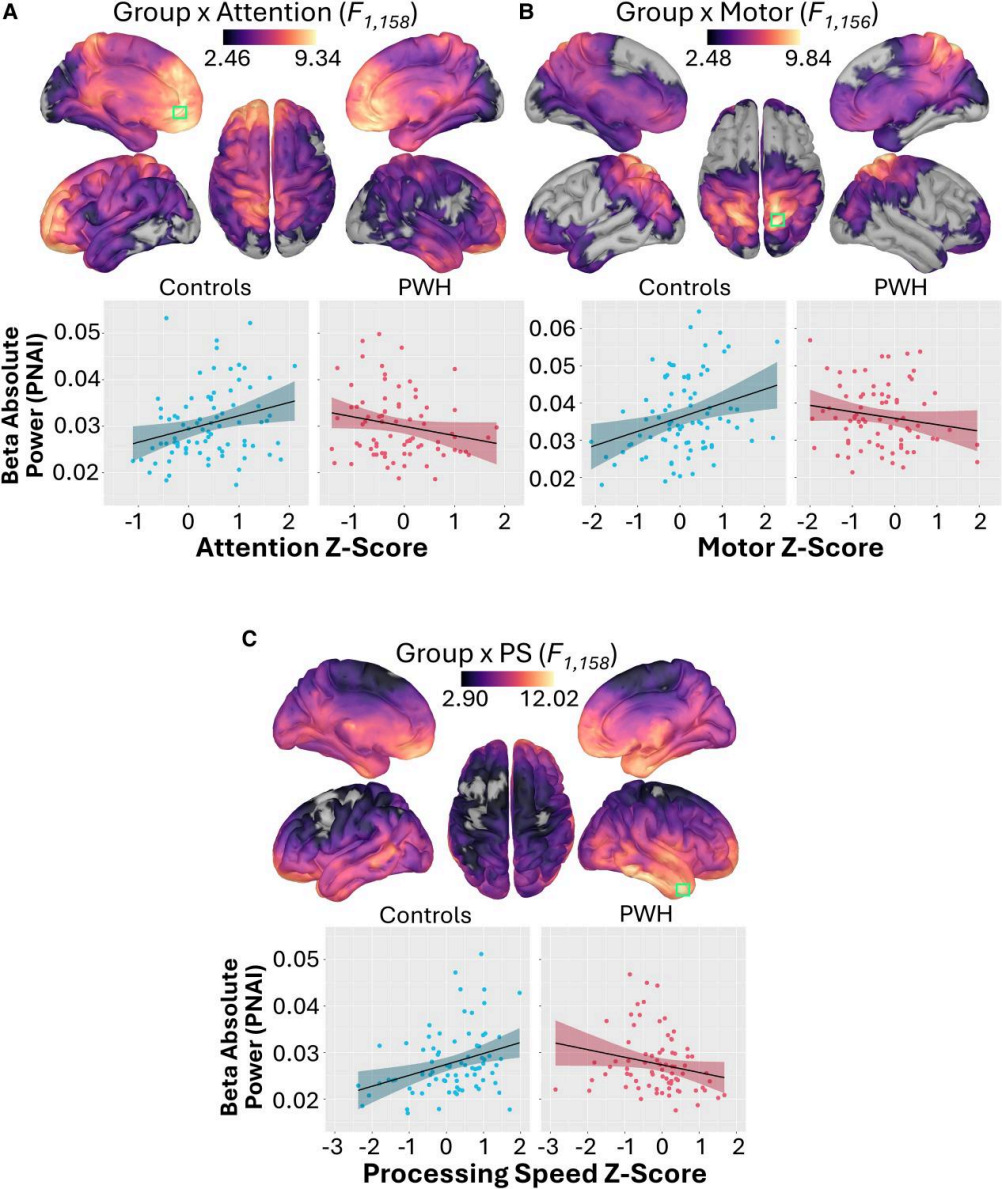
**The moderating effect of serostatus on the relationship between resting-state spontaneous beta power and cognitive composite scores.** Cortical surface maps (top) display the vertex-wise *F*-values representing the moderating effect of HIV on the relationship between spontaneous beta activity and the attention (**A**), motor (**B**) and processing speed (**C**) domains. Each of these moderating effects was calculated using a multiple regression. The green box indicates the vertex containing the strongest effect. For illustrative purposes, each participant's absolute power is plotted against the *Z*-scores for each cognitive domain (bottom), separately for controls and PWH. The least squares line is plotted in black, and the shaded region illustrates the 95% confidence interval. For attention, the moderation effect was particularly strong in the anterior cingulate cortex (*F*_1,158_ = 9.38, *P*_fwe_ < 0.01). For motor, this effect was strongest in the superior parietal lobule (*F*_1,156_ = 9.84, *P*_fwe_ < 0.01), whilst the strongest effects for processing speed were seen in the anterior temporal and prefrontal cortices (*F*_1,154_ = 12.02, *P*_fwe_ < 0.01).

For the attention domain, the moderating effect of HIV was observed on beta power in two bilateral clusters. This effect peaked in the anterior cingulate (*F*_1,158_ = 9.38, *P*_fwe_ < 0.01) and extended across the frontal and parietal lobes, as well as the superior temporal cortices (Fig. 4A). Follow-up testing revealed a positive relationship between attention and beta power in the control group (*t*_81_ = 2.50, *P* < 0.05), whereas no effect was observed in PWH.

Regarding the motor composite, the moderating effect of serostatus was observed on beta power across the bilateral parietal lobe, superior occipital areas and the anterior prefrontal cortices, with a peak in the right superior parietal lobule (*F*_1,156_ = 9.84, *P*_fwe_ < 0.01; Fig. 4B). Follow-up analyses showed a positive relationship between beta power and motor scores in the control group, such that those with stronger beta power performed better (*t*_81_ = 2.86, *P* < 0.01). No effect was observed in PWH (*t*_75_ = −1.52, *P* > 0.05).

As per processing speed, a moderating effect of HIV was found on beta power across all lobes of the brain bilaterally, with a peak in the right temporal pole (*F*_1,154_ = 12.02, *P*_fwe_ < 0.01) and sub-peaks in left temporal pole and the prefrontal poles bilaterally (Fig. 4C). Follow-up testing revealed a positive relationship between beta power and processing speed in controls (*t*_78_ = 3.08, *P* < 0.01), whereas no relationship was observed in PWH (*t*_78_ = −1.91, *P* > 0.05).

### Impact of HAND on the relationship between spontaneous activity and cognitive performance

To determine whether the relationship between spontaneous activity and cognitive function differed between cognitively impaired (i.e. those with HAND; *n* = 20) and unimpaired PWH (*n* = 59; see Table 2), we submitted the correlation coefficients between the composite *Z*-score per domain and spontaneous activity for each sub-group to a Fisher's *z*-test, separately for each of the three significant serostatus by cognitive performance interactions on beta power (see the ‘Effect of HIV on the relationship between cognitive performance and spontaneous activity’ section ). Here, the spontaneous beta activity was taken from the vertex showing the strongest interaction. Of these tests, only the relationship between processing speed and beta power in the anterior temporal lobe reached significance (*z* = −2.28, *P* < 0.05). Follow-up analyses showed that processing speed and beta power were negatively related for unimpaired PWH (*r*_57_ = −0.45, *P* < 0.001), but not related in those with HAND (*r*_18_ = 0.14, *P* > 0.05).

**Table 2:** Demographics and neuropsychological profiles for HIV patients with and without HAND

		HIV with HAND (*n* = 20)	HIV without HAND (*n* = 59)	*P*-value
**Demographics**	**Age (years)**	50.969 (13.524)	46.076 (12.704)	0.147
	**Sex assigned at birth (*n*)**	17 male/3 female	44 male/15 female	0.337
	**Race (*n*)**	14 White/2 Black/1 Asian/0 American Indian/Alaska Native/3 multiracial	47 White/7 Black/2 Asian/1 American Indian/Alaska Native/2 multiracial	0.427
	**Ethnicity (*n*)**	15 not Hispanic/4 Hispanic/1 NA	53 not Hispanic/5 Hispanic/1 NA	0.251
	**Education (years)**	12.8 (2.966)	14.241 (2.179)	0.023
**HIV clinical metrics**	**CD4 nadir (cells/µl)**	156.556 (149.935)	272.358 (227.726)	0.048
	**Current CD4 count (cells/µl)**	757.471 (493.669)	789.906 (332.923)	0.758
	**Current viral load (copies/ml)**	<50^a^	<50^a^	
	**Years since HIV diagnosis**	18.4 (10.364)	12.797 (7.932)	0.758
	**Years on ART**	14.632 (8.946)	11.561 (7.672)	0.152
**Neuropsychological performance**	**Learning *Z*-score**	−0.484 (1.055)	0.575 (0.78)	<0.001
	**Memory *Z*-score**	−0.364 (1.04)	0.541 (0.713)	<0.001
	**Processing speed *Z*-score**	−0.825 (0.891)	0.124 (0.655)	<0.001
	**Attention *Z*-score**	−0.669 (0.519)	0.083 (0.678)	<0.001
	**Executive function Z-Score**	−0.597 (0.926)	0.342 (0.783)	<0.001
	**Language Z-Score**	−0.613 (1.015)	0.171 (0.803)	0.001
	**Motor Z-Score**	−0.818 (0.622)	−0.163 (0.851)	0.002

Means and standard deviations are displayed for each continuous variable. Differences in mean age between males and females were assessed using an independent samples *t*-test; differences in race, ethnicity and handedness were assessed using *χ*^2^ tests. *P*-values for neuropsychological performance variables are FDR corrected.

ART, antiretroviral therapy; HAND, HIV-associated neurocognitive disorder; NA, not answered.

^a^Participants with a viral load of >50 copies/ml were ineligible to participate in this study.

## Discussion

The current study examined whether spontaneous cortical activity during rest differed in PWH compared with controls and whether certain patterns of spontaneous activity were differentially related to particular cognitive domains amongst PWH and controls. First, we found that PWH scored significantly lower on the attention, memory, learning and motor domains of cognitive function compared with controls. Regarding neural data, we found that spontaneous cortical activity in the gamma band was elevated in PWH in the inferior parietal, superior temporal and prefrontal cortices. In addition, HIV status moderated the relationship between spontaneous beta activity and the attention, motor and processing speed domains. These moderating effects tended to peak in association cortices involved in complex cognitive processes, including regions serving multimodal integration and top-down signalling of low-level neural processes. Importantly, each interaction was characterized by a significant association between cortical activity and cognitive assessment in controls but not PWH, suggesting that spontaneous activity in cortical regions associated with specific cognitive domains was altered in PWH. In other words, the typical neural processes that improve cognitive performance were absent in those with HIV. Taken together, these findings are broadly consistent with previous findings that indicate system-level brain dysfunction associated with HIV infection^23^ and provide novel data extending observations of elevated gamma activity to the resting state.

One of our most important findings was the elevated spontaneous gamma power in PWH, which was observed across bilateral peaks that included the parietal, temporal and prefrontal cortices. Previous work has shown elevated gamma power in the somatosensory cortices of PWH during the pre-stimulus baseline period of a somatogating task,^19^ as well as in visual cortices during a visual attention task,^22^ and the prefrontal cortices.^20^ Interestingly, the current data showed elevated spontaneous gamma in the somatosensory and prefrontal but not visual cortices, thus replicating previous studies of the somatosensory system^18,19,69^ whilst disagreeing with that of the visual system.^22^ These findings may indicate that the alterations in visual cortices are more task dependent and reflect deficits in the rate at which gamma oscillatory activity returns to baseline during visual processing tasks. In other words, based on the available data, spontaneous gamma activity in visual cortices may not be elevated in PWH, but instead power levels may appear elevated during the pre-stimulus baseline of visual attention tasks because the gamma visual response from the previous trial has not fully dissipated in PWH. Future neurophysiological studies collecting both resting-state and visual task-based data are needed to distinguish these alternatives. More broadly, spontaneous gamma power is known to be heightened in healthy older adults^42^ and has been tied to age-related declines in somatosensory function.^70^ Such increases in spontaneous gamma have been linked to dysfunction in inhibitory GABAergic circuitry,^71-74^ which is thought to be particularly vulnerable to the persistent neuroinflammation that can accompany HIV infection.^75,76^ Taken together, elevations in spontaneous gamma activity amongst PWH may be a critical marker of brain dysfunction in these individuals.

Whilst overall group differences were observed for gamma activity, interactions between HIV serostatus and cognitive assessment scores were observed only in the beta frequency band. Specifically, controls, but not PWH, showed stronger spontaneous beta activity with better performance on cognitive assessments. Beta activity has long been considered a signature of increased cortical processing across the brain and has been tied to several functions including visual attention,^77-79^ motor behaviour^40,80-82^ and higher-order cognition.^83,84^ Along these lines, the beta rhythm has been shown to be a signature of efficient cortical processing^85^ and supports the transmission of information across distant cortical locations.^86^ Interestingly, similar to gamma activity, the emergence of cortical beta rhythms likely depends on GABAergic transmission.^87-89^ In the context of this literature, the current results suggest that HIV may impact cognition *vis-à-vis* a disruption of the cortical beta rhythm, and future work should continue to explore the extent to which HIV impacts GABAergic neural communication.

The interactions for both the attention and motor domains involved beta activity in similar brain regions. For attention, the effect was most prominent within the anterior cingulate cortex, with sub-peaks in superior parietal cortices. For motor, the effect was conversely strongest in superior parietal cortices, with sub-peaks in the anterior cingulate cortex. Both regions have been consistently recognized as critical nodes in visual attention processes.^90-93^ Along these lines, increased spontaneous beta activity, especially over the frontal cortex, has long been associated with increased cortical processing.^94,95^ The anterior cingulate cortex, in particular, is thought to play a role in maintaining attention toward behaviourally relevant information,^92^ whilst the superior parietal is similarly involved in top-down attention^96-98^ but also in the initial orienting of attention.^99,100^ Interestingly, activation of the superior parietal cortex has also been observed during motor imagery^101,102^ and planning,^103^ suggesting that it plays a role in mediating the relationship between perceptual and motor functions.^104,105^ Thus, whilst these regions are broadly recognized for their role in visual attention, their involvement in the motor domain is also well supported. Moreover, motor behaviour has long been tied to beta oscillatory activity patterns in the motor cortex,^40,80-82^ which previous works have shown to be abnormal in PWH.^106^ Such beta aberrations in the motor cortex of PWH were recently tied to dysfunction in the mitochondrial redox environment.^107,108^ In the context of previous literature outlining the function of the anterior cingulate cortex and superior parietal cortex, our findings of aberrant spontaneous beta activity may reflect HIV-related dysfunction of the systems-level cortical circuits underlying attention and motor planning, as well as their integration.

We also found that HIV status moderated the relationship between spontaneous beta activity and processing speed across widespread cortical regions. This effect was strongest in the temporal pole, which is thought to drive semantic object recognition^109^ by coordinating incoming sensory information and memory cues along the ventral pathway.^110^ Whilst this interaction indicated better processing speed with stronger beta amongst controls, but not PWH, processing speed overall did not differ between controls and PWH. Thus, these results suggest that similar levels of processing speed may involve different neural circuits in PWH and healthy controls. This interpretation is consistent with task-based findings where otherwise normally performing PWH show altered, perhaps compensatory, patterns of induced brain activity.^24,25,27,30,111^ Interestingly, cognitively impaired PWH (i.e. those with HAND) showed a different relationship between beta activity and processing speed than unimpaired PWH, suggesting further that spontaneous beta activity may be particularly important to the processing speed deficits observed in those with significant impairments across multiple domains of function. Given the broad impacts of processing speed on activities of daily living and overall cognition, it could also be that spontaneous beta levels indirectly impact other domains in cognitively impaired PWH, but more work is needed to identify the specific relationships.

Regarding the overall functional significance of our findings, our data in controls suggest that the health or functionality of brain circuits underlying specific cognitive domains can be assessed without a task by focussing on the power of region-specific and spectrally specific spontaneous cortical activity. Specifically, we found that spontaneous beta levels were coupled to neuropsychological performance metrics across several domains in controls. Whilst this finding is not entirely surprising, it is novel, and future work should further probe this relationship. Regarding PWH, spontaneous levels did not appear to be related to neuropsychological performance. Although speculative, we propose that many of these participants would have adequately performed cognitive tasks during the MEG imaging by recruiting other brain regions to compensate for dysfunction in the core circuitry. For example, previous studies have found that PWH show stronger activation of frontal cortical regions during visual attention^27^ and working memory tasks,^25,111,112^ pointing to a heightened, compensatory recruitment of cognitive resources.^113^ We think this is especially likely as past normative studies have shown that elevated spontaneous levels are associated with reduced neural oscillations and behavioural performance.^114,115^ However, caution is warranted as this interpretation is only speculation given our MEG recordings occurred during a task-absent rest condition. Future work should directly test if altered spontaneous cortical activity predicts task-induced activation increases of other, compensatory, brain regions in PWH.

Before closing, several limitations should be noted to qualify the current results, which may be addressable in future work. First, as touched upon above, despite the use of neuropsychological testing, the extent to which resting brain activity can be tied to behaviour and cognitive function is limited. Future work should continue to tie aberrant brain activity to different behavioural tasks in the context of functional imaging. In addition, future studies should carefully consider the use of eye-open and eye-closed rest conditions, given the known differences in brain activity especially in visual cortices.^116,117^ The goal of the current study was to compare brain activity between controls and PWH, and we did not consider the effects of individual differences in HIV clinical metrics (e.g. length since diagnosis and length since starting ART) on brain function. Along these lines, the current study did not investigate the effect of chronological age given that the neuropsychological composite *Z*-scores were already age adjusted. Typical age-related differences in cognition may interact with these HIV factors, perhaps due to accelerated biological ageing.^30,33,43,44^ Future studies should consider these factors to carefully delineate the effect of HIV on the brain from those associated with typical healthy ageing.

To summarize, the current results indicate that spontaneous gamma power is elevated in PWH across regions of the inferior parietal, prefrontal and superior temporal cortices. Spontaneous beta activity differently predicted performance on the attention, motor and processing speed domains of cognitive function in PWH and controls. In each case, controls showed a relationship between spontaneous beta activity and cognitive function, whilst PWH did not. These results suggest that spontaneous beta and gamma rhythms are impacted by HIV, perhaps through a disruption of GABAergic signalling. More generally, these results are consistent with previous work showing that HIV infection impacts multiple brain regions and provide important new data on how spontaneous cortical rhythms differ in PWH versus people without HIV.

## Data Availability

The data used in this article will be made publicly available through the COINS framework at the completion of the study (https://coins.trendscenter.org/). Data processing pipelines followed previous studies^118^ using a combination of Brainstorm,^119^ which is documented and freely available for download online under the GNU general public license (http://neuroimage.usc.edu/brainstorm), and CAT12^56^ toolboxes.
